# Students’ Intention of Visiting Urban Green Spaces after the COVID-19 Lockdown in China

**DOI:** 10.3390/ijerph18168601

**Published:** 2021-08-14

**Authors:** Jiayi Liu, Zhikai Peng, Xiaoxi Cai, You Peng, Jiang Li, Tao Feng

**Affiliations:** 1 School of Architecture and Art, Central South University, Changsha 410083, China; liujiayi@csu.edu.cn (J.L.); lijiang@csu.edu.cn (J.L.); 2 Department of Architecture, University of Cambridge, Cambridge CB2 1PX, UK; zp254@cam.ac.uk; 3 College of Art and Design, Hunan First Normal University, Changsha 410205, China; 4 Urban Planning and Transportation Research Group, Department of the Built Environment, Eindhoven University of Technology, P.O. Box 513, 5600 MB Eindhoven, The Netherlands; t.feng@tue.nl; 5 Graduate School of Advanced Science and Engineering, Hiroshima University, Hiroshima 739-8527, Japan

**Keywords:** COVID-19, theory of planned behavior, perceived knowledge, risk perception, structural equation modeling, urban green space

## Abstract

This study addresses students’ perceptions of using urban green spaces (UGSs) after the easing of COVID-19 lockdown in China. We questioned whether they are still mindful of the risks from the outdoor gathering, or conversely, starting to learn the restoration benefits from the green spaces. Online self-reported surveys were distributed to the Chinese students aging from 14 to 30 who study in Hunan and Jiangsu Provinces, China. We finally obtained 608 complete and valid questionnaire forms from all participants. Their intentions of visiting UGSs were investigated based on the extended theory of planned behavior model. Structural equation modeling was employed to test the hypothesized psychological model. The results have shown good estimation performance on risk perception and perceived knowledge to explain the variances in their attitudes, social norms, and perceived behavior control. Among these three endogenous variables, the perceived behavior control owns the greatest and positive influence on the behavioral intention, inferring that controllability is crucial for students to make decisions of visiting green spaces in a post-pandemic context.

## 1. Introduction

A novel coronavirus disease (COVID-19) has received worldwide attention since its first emergence in Wuhan at the end of 2019. The COVID-19 outbreak was then drastically escalated as a pandemic [1,2]. As of March 2021, no fewer than 115,250,000 COVID-19 cases had been confirmed in the world, including 2,560,000 deaths according to the statistics released by the World Health Organization. The considerable transmission brought severe public health crises for almost every country due to its hyper infectivity [3]. According to clinical evidence, respiratory droplets and personal contact are the primary transmission channels [4]. Given its nature of asymptomatic transmission, people are advised to be precautions to protect themselves and others, such as keeping social distancing, frequent hand washing, wearing face coverings in public, etc. [5]. Many national governments also issued a variety of policies and guidelines to require people to stay at home for protecting the national health institutes from service overload as the pandemic escalated [6].

Under the first wave of COVID-19, students had experienced uncertainty in their academic success, future careers, and social life change [7]. The uncertainty and its bearings on students’ academic progress could also influence students’ psychological well-being [8,9,10]. Students have been found more vulnerable in combating mental stress than the general population even before the pandemic, which increased varying degrees of psychosomatic problems, such as anxiety, burnout, depressive moods, lack of self-esteem, substance abuse, and suicidality [11,12,13,14]. Studies have reported high detection rates of mental problems in Chinese adolescents and students, ranging from 5.9% to 10.7% [15,16]. Most of the educational institutions around the world were shut down or transformed to online education during the pandemic, leading to great disruption in students’ study and outdoor activities [17,18]. Studies showed that longer durations of quarantine were associated with poorer mental health, avoidance behaviors, and anger [19,20,21]. A study on the psychological pressure of Chinese students during the COVID-19 pandemic found that out of 7143 students studied, 0.9% had severe anxiety, 2.7% had moderate anxiety, and 21.3% had mild anxiety [22].

In recent years, interest has grown in the positive benefits that might be gained from natural environments and time spent outdoors [23,24,25]. Nature exposure, including park playing, bird watching, and sporting activities in natural environments can keep people mentally active and healthy [26,27,28]. However, restrictions on the use of public green spaces, quarantine, and social distancing as effective measures implemented to tackle the COVID-19 and protect public health during the pandemic. Countries across the world have introduced policies such as stay-at-home lockdowns, restrictions on public events, social gatherings, and public transport, and the closure of schools. Students had no chance to conduct leisure activities in public spaces. During the post-pandemic era, the role of public green space may become important for mental recovery [29,30].

The purpose of this study is to estimate the impact of the COVID-19 on students’ mental health and their decisions to revisit UGSs after the easing of the lockdown. Moderated by their attitudes, social norms, and perceived control, their motivations and reluctance upon the traveling decision were proposed for measurement. We utilized the theory of planned behavior (TPB) model in environmental behavioral studies [31,32,33] and extended the structure of the basic TPB model by adding two additional components, risk perception, and perceived knowledge, which enabled us to access students’ decisions on visiting outdoor UGSs.

In this regard, we intend to (1) predict the impact of the COVID-19 pandemic on students’ perception of using the greenspaces after the COVID-19 lockdown; (2) test the validity and reliability of each hypothesis; (3) examine the estimation performance of the extended TPB model; (4) inform schools and parents of the restoration benefits from visiting UGSs and convince relevant administrative agencies that the UGSs can satisfy students with a variety of needs.

## 2. Literature Review

In recent years, many studies have explored the pandemic impact on individual mental health and their behavior pattern change. During the SARS epidemic, Hawryluck et al. (2004) pointed out a high incidence of psychological distress among quarantined individuals in Toronto [19]. Saadatian et al. (2010) put forward a self-report approach to evaluate how much the knowledge of infectious diseases would contribute to alleviating anxiety in the USA, showing that 20% of respondents familiar with avian influenza had changed their travel behaviors during the epidemic [34]. Novelli et al. (2018) studied the Ebola-induced tourism crisis in Gambia [35]. Bi et al. (2019) examined how the epidemic prevalence and the information on community behavior-change affect people’s emotions [36]. A psychological study found that 53.8% of respondents rated “moderate” or “severe” when asked about the pandemic disturbance in their daily lives, while 16.5% have reported moderate to severe depressive symptoms [37]. Another study has demonstrated the biggest change in people’s daily life is to avoid visiting crowded places as much as possible [30].

University students make up a significant part of mental-health victims due to the one-size-fits-all lockdown regulations in China [38,39,40]. The lockdown had led to the development of irregular daily routines when students are asked to self-isolate in their dormitory buildings, which undermined their mental health to a large extent. There was evidence in the past showing that students who are more likely to experience anxiety, depression, and emotional fluctuations may, in turn, affect their plans of behavior during the epidemic period. For example, some students must reduce social activities to avoid poor sleeping quality induced by psychological stress [36,41,42,43]. Odriozola-González et al. (2020) had sampled the data of psychological well-being of Spanish university students during the COVID-19 pandemic and found 34.19% of respondents showed moderate to extremely severe depression symptoms, 21.34% showed extremely severe anxiety symptoms, and 28.14% had exhibited moderate to extremely severe stress symptoms [44]. A list of key stressors has been identified to cause anxiety among students during the lockdown, such as the lack of financial support, unexpected educational disruptions [22], the uncertainties in their future career paths [8,45], and the massive spread of astounding and inaccurate news in the social media [46].

Seeking solutions for mental restoration, researchers have demonstrated the benefits gained from natural environments and time spent outdoors [2,23,24]. There is increasing evidence concerning the positive effects of frequent exposure to natural environments [28,47,48]. For example, some have found that the increase of views toward nature can restore people from mental stress and fatigue and potentially improve their health and well-being [49,50], and this gives rise to additional benefits, such as attention restoration, sense of connectedness, companionship, and self-esteem [51]. The biodiversity and sports infrastructures contribute to positive experiences in UGSs. Taking notice of the wildlife and physical exercises are both important aspects of keeping physically healthy [2,26,27]. In the education sector, studies have highlighted the importance of nature and outdoor activities to students [18,52,53,54]. Compared to indoor settings, frequent engagements in nature will contribute to the alleviation of tension, confusion, anger, and depression. A survey among 523 American students by Coon et al. (2011) has demonstrated a strong relationship between students attending outdoor activities and the likelihood of efficient mental recovery. They have also discussed the approaches to nudge positive attitudes towards physical exercise [27]. Another study in Canada has investigated student’s activities in the natural environment and found its association with inhibiting positive emotions [55].

In the context of COVID-19, visiting UGSs has been greatly compromised even after the easing of the lockdown in most areas in China. This phenomenon requires a deeper understanding of the reasons behind the travel intention to these restorative places. Through reviewing the travel intention-related literature, we have located TPB, one of the widely practiced theories in social psychology, which aims to predict behavioral decision-making [31]. The high applicability of the TPB model has been proved for studying a variety of human behavior, especially in education [56], tourism [57], consumption behavior [58], environmental protection [59], etc. It is also widely used by epidemiological studies on the relationship between individual decision making and consequential behavior during the vast spread of contagious diseases. For example, Prasetyo et al. (2020) evaluated people’s perception of the preventive measures during the lockdown of COVID-19 by integrating the protection motivation theory (PMT) and the TPB model [60], which explained the behavioral intention of wearing face coverings during the spread of seasonal influenza. Moreover, the TPB model is considered efficient in predicting behavioral change such as panic buying due to the uncertainty of the lockdown [61], the habituation of frequent hand sanitization [62], the changes in dining behavior [63], and the choice of safer travel destinations during the epidemic. In addition to estimating the changes in behaviors patterns, the TPB model can also quantify the causal effects of the motivation for vaccination [64,65].

The three principal components of TPB are the attitude toward the behavior, the subjective norm, and the perceived behavioral control [31]. However, the basic TPB model requires further strength in its explanatory adequacy. Previous findings indicated that other potential predictors, such as perceived knowledge and risk perception can optimize the basic TPB model with only three predictors [66,67]. They also contribute to directing behavioral decision-making during epidemics [19,34,61,62]. Other studies have revealed that the level of knowledge about COVID-19 will affect people’s perceived vulnerability and their behavior [60]. The risk perception has shown a significant correlation with the preventive health measures during the epidemic [68], primarily taken by the governments, people at workplaces, and family members [42]. The ongoing COVID-19 situation has left a tremendous shadow on students who are overly cautious of the health risks when planning to be outside [69,70]. Cao et al. (2020) investigated 7143 Chinese college students during the period of COVID-19 and revealed students’ anxiety varying in severe, moderate, and mild levels. When people sense the risks from their travels before departures, their plans are more likely to be put off or canceled [71]. Based on these previous findings, we have hypothesized that students’ perceived knowledge and their risk perception of COVID-19 would drive their subjective norms and behavioral attitudes, respectively, and that the explanatory adequacy on their travel intentions would increase by including perceived knowledge and risk perception in the basic TPB model.

## 3. Research Questions and Hypotheses

### 3.1. Theory of Planned Behavior

We question the key psychological moderators that significantly affect students’ perception of visiting UGSs after the easing of the COVID-19 lockdown. Their motivations and hesitancies upon the travel decisions are critical to their behavioral intentions, as well as the actions on making the visits. The stronger their motivations are, the more likely they would take the actions [72]. According to the TPB model, individual behavioral actions are directly influenced by behavioral intention, which can be measured by their attitude, subjective norm, and perceived behavioral control, three key psychological constructs in our study contexts.

Attitude is the first psychological construct in the TPB model associated with the travel decisions by the individuals, which measures the degree of the positive or negative tendencies toward the target behavior and can also effectively predict the frequency of behavioral intentions [73]. According to the literature, forming a positive attitude towards visiting green spaces is based on the assumption that the natural environment would afford sufficient green exposure and alleviate the anxiety and loneliness of travelers, which, in turn, gives rise to the arousal of positive perceptions [73].

Subjective norms are defined as those beliefs of what important rules to comply with, and about the behavior that other persons or parties would encourage you to follow or not [74]. In this study, we attempt to test if the subject’s intention of visiting the greenspaces will change due to any social norms or pressure, for example, the discouragement from other individuals or social relations, e.g., his or her own family, classmates, friends, and neighbors, etc.

The third psychological construct in the TPB model is the perceived behavior control. It assesses the subject’s perception concerning how difficult the target behavior could be handled and performed, or whether he is able to participate in the target behavior. In the context of this study, if students hesitate to visit green spaces, a trading-off process would occur in their minds on whether the resources such as time and energy are enough to make their trips happen. The interpersonal differences of the perceived behavioral controls will either facilitate or hinder their travel intentions.

Therefore, five hypotheses are proposed in line with the basic TPB model as follows:

**Hypothesis** **1** **(H1).** *Students’ attitude (ATT) influences behavioral intention (BI) of visiting UGSs*;

**Hypothesis** **2** **(H2).** *Students’ perceived behavior control (PBC) influences BI of visiting UGSs*;

**Hypothesis** **3** **(H3).** *The subjective norms (SNs) of students influence BI of visiting UGSs*;

**Hypothesis** **4** **(H4).** *ATT influences PBC*;

**Hypothesis** **5** **(H5).** *SNs influence PBC*.

### 3.2. Expanding the Theory of Planned Behavior

The basic TPB model comprises only three principal psychological constructs that are inadequate to cover other contributory factors such as the perceived risk and knowledge. Nevertheless, the basic TPB model provides an adaptable framework for various research backgrounds. Ajzen also claimed that the basic TPB is a user-friendly model allowing further expansion of the model to meet a variety of research needs [31]. Therefore, we incorporated two additional moderators in the model-risk perception and perceived knowledge, which have been already tested in other studies in the literature [57,75,76].

Firstly, risk perception is closely related to subjective norms. Studies have revealed that risk perception has significant negative effects on individuals’ decision-making processes and their consequential behaviors [76,77,78,79]. Choosing a destination with less perceived risk will have a positive influence on tourists’ behavioral intention and confidence in making their travels [80]. In comparison with other social groups, students are more cautious about their health-related risks when going out. In the context of COVID-19, risk perception is also associated with government measures, people at their workplaces, personal acquaintances, and other social relations [42,68].

Secondly, we added another variable-perceived knowledge moderating one’s behavioral attitude. The psychologist Kaplan once pointed out that gaining the knowledge of a problem will significantly affect a person’s decision making [81], which was also empirically validated in the area of tourism [81,82]. In this study, the perceived knowledge of COVID-19 has been specified as students’ familiarity with the knowledge of the COVID transmission channels and the preventative methods against catching the disease.

Therefore, we added two hypotheses regarding perceived risk and knowledge, following the previous five hypothetical paths in the basic TPB model.

**Hypothesis** **6** **(H6).** *Risk perception (RP) impacts SN*;

**Hypothesis** **7** **(H7).** *Perceived knowledge (PK) impacts ATT*.

**Figure 1:**
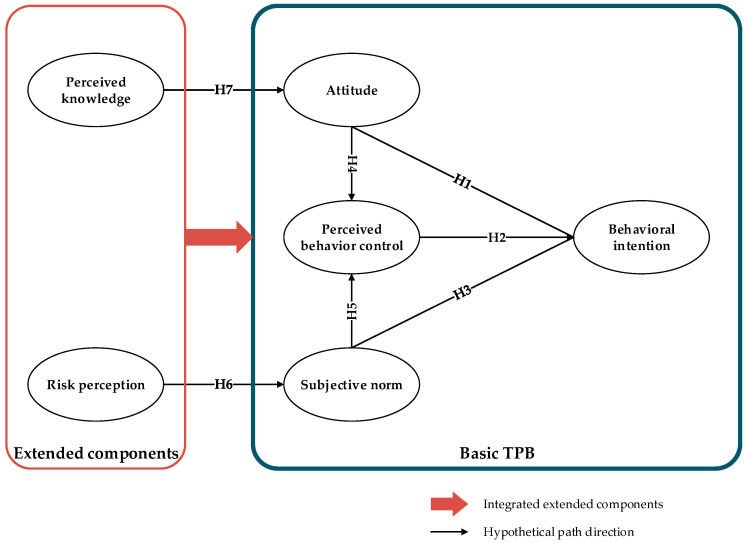
Diagram of the extended TPB model.

To recapitulate the above definitions of our seven hypotheses, we built the extended TPB model to study student’s intention of visiting UGSs after the easing of COVID-19 lockdown (see Figure 1).

## 4. Methodology

### 4.1. Data Collection

The online questionnaire was designed based on the basic TPB model [31,59,83]. In total, 25 question items were included in the online questionnaire, of which the first part is related to respondents’ demographical characteristics, socioeconomic status, dwelling conditions, and the frequency of visiting UGSs. The second part of the questionnaire is made up of five principal constructs of items for collecting the data of respondents’ attitudes, subjective norms, perceived behavior control, the COVID-19 related knowledge, and their risk perception while visiting UGSs. These five psychological constructs were evaluated with two or three question items on a Likert scale from 1 (strongly disagree) to 7 (strongly agree). The order between question items was slightly modified according to the feedback from the small samples test before the mass online distribution. The online data collection was finished before October 2020 mostly for Chinese students studying in Hunan and Jiangsu Provinces, China. The qualified respondents are required to be elder than 10 years and have the UGSs experience for at least one time in the past 3 years. The respondent spent about 9 min on average completing the questionnaire (see Table A1 Appendix A). Finally, we obtained 608 effective samples, which was considered sufficient to conduct data analysis [84,85].

### 4.2. Structural Equation Modeling

Structural equation modeling (SEM) is a general statistical modeling method widely used in social and behavioral science to test and evaluate multiple causalities [86,87,88]. It is generally expressed by a linear equation system, divided into a measurement model and a structural model. The measurement model reflects the relationship between the potential variables and the observed variables, and the former can be defined by the combinations of the latter. The structural model provides an estimate of the relationship between each latent variable. The specific expression of the model is as follows:

(1) $$X=\Lambda_{x}\xi +\delta$$

(2) $$Y=\Lambda_{y}\eta +\varepsilon$$

(3) η=Βη+Γξ+ζ

where Formulas (1) and (2) are the expression of the measurement model, X is the vector composed of exogenous variables, and Y is the vector composed of endogenous variables; $\Lambda_{x}$ is the factor load matrix of exogenous variables on exogenous latent variables, and $\Lambda_{y}$ is the factor load matrix of endogenous variables on endogenous latent variables; ξ is exogenous latent variables and η is endogenous latent variables; δ and ε are the measurement standard errors of exogenous variable X and endogenous variable Y, respectively. Equation (3) is the structural model expression, Β and Γ represent the relationship matrix between endogenous latent variables and the influence matrix of exogenous latent variables on endogenous latent variables, and ζ is the unexplained part of η in the model.

A couple of goodness-of-fit indexes have been used in this study to examine how well the hypothesized model fits the observation datasets. The root means squared error of approximation (RMSEA) [89] is a measure of the estimated discrepancy between the actual population and the model-implied population, scaled by the number of degrees of freedom in covariance matrices. According to the literature [90], the model fit is considered acceptable when its RMSEA value is ranging from 0.05 to 0.08, compared to a satisfactory model fit with that less than 0.05. Secondly, the discrepancy between the observation and hypothesized model is examined by the comparative fit index (CFI), which provides a measure of complete covariance in the data ranging from 0 to 1. The advantages of CFI lie in the ability to penalize complex models without affecting the sample size. The higher CFI value indicates a better fit. A satisfactory fit requires a reference value of CFI no less than 0.90 [91]. The range of incremental index (IFI) and Tucker–Lewis index (TLI) is between 0 and 1, and the closer it is to 1, the better is the fitting. Generally, a statistical result with these two values greater than 0.9 indicates that the model fits the observed data well.

## 5. Results and Discussion

### 5.1. Descriptive Statistics

**Figure 2:**
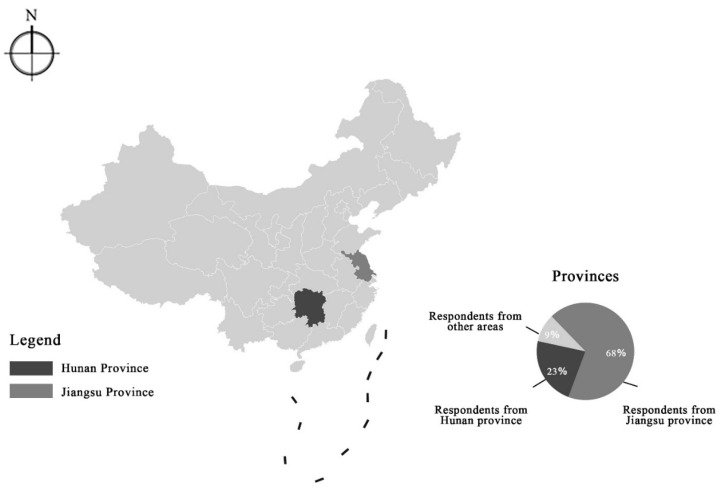
Locations of online survey participants.

The sociodemographic characteristics of the respondents (N = 608) are shown in Table 1. Accordingly, 55.92% (N = 341) are male respondents and 44.08% (N = 268) are female. Most of them are younger than 35 years old with an average age of 17. About 55.05% of the respondents have achieved an undergraduate degree or above, followed by the group of high school graduates (42.68%). As for their ethnic backgrounds, the majority were made up of Han (95.58%) (see Figure 2). Regarding the frequency of visiting, 6.44% went to UGSs almost every day, whereas 12.12% made their travels every week, and the rest of the vast majority occasionally visited the UGSs in the past year (56.69%).

### 5.2. SEM Estimation Results

We utilized a maximum likelihood estimation (MLE) algorithm in the SEM process to assess the estimation performance of the hypothesized model based on the theory of planned behavior. As shown in Table 2, the indexes for goodness to fit indicate that the test results are significant.

**Figure 3:**
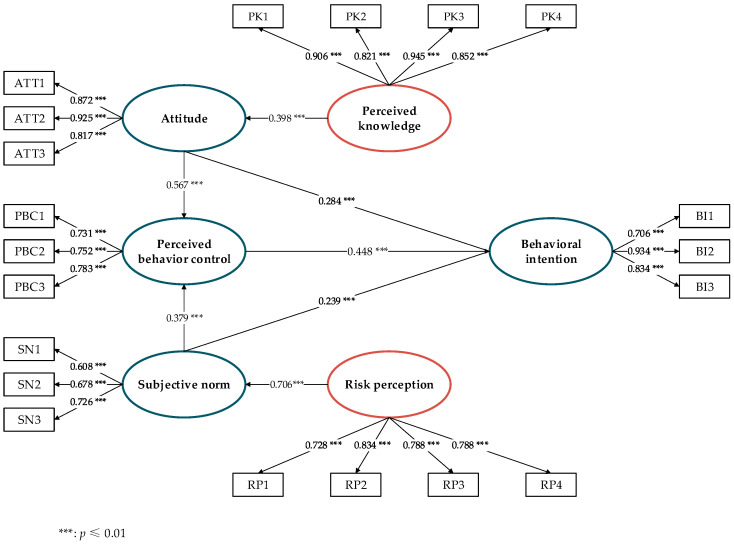
Diagram of SEM estimation results.

The estimation results by the SEM have been integrated into Figure 3. Three hypothesized paths toward the behavioral intention were assessed in the central, originating from the attitudes, the subjective norm, and the perceived behavioral control. The loadings between all measured variables and corresponding latent constructs demonstrate significant causal effects (see Table 3). Seven hypothetical paths (see Table 4) have shown good estimation performance, inferring that attitude, subjective norms, and perceived behavioral control have a positive impact on students’ behavioral intention to visit green spaces after the easing of the COVID-19 lockdown. Therefore, H1, H2, and H3 are verified.

Among the three principal constructs, the perceived behavioral control has the greatest influence on behavioral intention, suggesting that controllability has a relatively strong impact on the decision making on risky events. If people know their ability to handle an event, their perceived behavioral control can effectively encourage or discourage their behavioral intention. Most respondents traveled to UGSs every day before the pandemic outbreak, which contributes to their control while making visits after the lockdown. Secondly, people may believe that the medical resources provided by the government are sufficient and reliable enough so that they would have a stronger sense of control over the whole process of traveling.

We also revealed a positive and significant effect of attitude on behavioral intention. The more positive the attitude toward the green spaces that students may have, the more motivated they will feel to make the visit. Prior to the pandemic, UGSs are known by students for its healing and restorative effects that significantly contribute to their stress recovery and to improving their ability of concentration [92,93,94]. After a long-term quarantine during the lockdown, many students desire to be back outdoors for any potential restorative and social opportunities, which forms a positive attitude. A previous investigation showed that having study breaks in green areas, especially larger ones, improved the well-being and cognitive performance of adolescents [25]. In recent years, with the increasing green area designated within and nearby the school teaching grounds [95,96], Chinese students have been benefited by using these pocked parks within walking distance. As an essential part of their daily routines at school, the positive experiences in these green, restorative places were added to their memory, which plays an incentive role in their intention to enjoy the green spaces again after the easing of the COVID lockdown.

Bock et al. (2005) had captured the link between subjective norms and behavioral intention [97]. Likewise, our research also proves that subjective norm is an important determinant of behavioral intention, yet weaker than that from attitude. Subjective norms are related to the social pressure and certain regulations to comply with, possibly from the communications and living experiences with their families and peers. Their plans of behaviors are also influenced by vast and dynamic social–cultural information through frequent verbal or behavioral interactions. In a study on the cultural influence on self-protection behavior during the H1N1 flu pandemic [98], Cho et al. (2015) found that subjective norms had a stronger power to predict the self-protection behavior in the Korean context than that in America, two countries typically known as individualistic and collectivistic national cultures. This implies that the local culture can have great influences on the formation of subjective norms. Living in a common culture of collectivism [99], Chinese students tend to live with the social norms everywhere from schools to homes. Most of them are willing to comply with the expectations of their families and peers to acquire positive feelings and a sense of belonging. Traveling to green spaces combines the risks from the social gathering of which the concerns vary among families situated in different physical and social environments. Other influences from social media are considered minor to their personal communities but still have obvious effects on their travel intentions. For example, many students mentioned in the questionnaires that after the pandemic, they are happy with visiting the nearest UGSs rather than the suburban forest parks. This result is consistent with the most up-to-date national traveling guidance and the disseminated information in Chinese mainstream social media, such as Weibo, TikTok, etc. [100].

The coefficients of the hypothetical paths H4 and H5 suggest that subjective norms have a positive impact on perceptual behavior control; the effect of attitude on perceived behavioral control is also positive and significant. This is consistent with previous studies that people with a more positive attitude would have a stronger ability of perceived behavior control toward a particular behavior [32,101].

The effect of risk perception on subjective norms and the effect of perceived knowledge on attitude have proved the validity of H6 and H7. They are associated with perceived behavior control and attitude in the extended TPB model. The perceived risk in an event will affect the controllability and their intention for taking action. The perceived knowledge serves as a long-term moderator in one’s attitude toward a behavior. Adequate knowledge of the pros and cons of visiting green spaces would enable rational thinking and mindsets, which positively guide the decision-making process.

## 6. Conclusions

This study aims to investigate students’ intention of visiting UGSs after the easing of the COVID-19 lockdown. We developed a holistic framework by extending the basic TPB model. Two additional variables—the perceived knowledge and risk perception—were incorporated in the basic TPB model made up of three focal psychological constructs—attitude, subjective norms, and perceived behavioral control. SEM was used to analyze the data collected online from Chinese students mainly in Hunan and Jiangsu Provinces. The statistical results proved the validity and reliability of seven hypothetical paths in the extended TPB model. Among three focal psychological constructs, perceived behavioral control shows the greatest and positive effect on behavioral intention, whereas attitude and subjective norms share similar factor loadings of estimation. The reliability of these three estimations on behavioral intention is stronger than those of the other four hypothetical paths, where risk perception owns the highest coefficient value to explain the perceived behavioral control. The findings suggest that controllability is vital in moderating the decision-making process, which balances the judgments between the foreseen benefits and risks in an event.

Meanwhile, this study urges that the related administrative agencies should pay particular attention to the physical and psychological well-being of Chinese students after the peak of the pandemic. Firstly, education institutions play a fundamental role in guiding students to cope with any pressing situations during the pandemic. They can provide remote counseling services to mitigate the mental distress of students, especially those isolated at home or in dormitory buildings. The education sectors and local authorities need to learn a variety of personal needs from student bodies. It is never too late to inform the general public of the restoration benefits from the limited natural resources in the urban area. For example, the local schools can hold lectures to inform students of the benefits of having daily exercise in the outdoor area nearby home, apart from the mainstream topics on the preventative measures against the COVID during the lockdown. More importantly, they should provide students with more opportunities to access UGSs kept in good control and management, such as regular disinfection, real-time monitoring, social distancing, and face coverings. The local planning authorities need to reconsider the ongoing massive development of large-scale green infrastructures. Conversely, they should spare the limited spatial resources for pocket parks and vertical greening [102,103], especially in hyperdense urban areas. There are still a number of students feeling the nearest green spaces are too far to access on foot. Reusing the leftover urban spaces and retrofitting them into small green spaces can effectively reduce the additional travel budget to UGSs and lower the risk perception of those students who do not live nearby large or medium green infrastructures.

There are limitations in this study and space for further improvement. First of all, the five psychological constructs connected by the extended TPB model are limited to encompassing all triggers of the behavioral intention, which could be either from the external environment or from the personal background. Another limitation lies in the lack of considering confounding factors. Other studies are suggesting that the emotional factors [66], perceived accessibility [104], and past behavior [59] also play important roles in the decision-making process of social and environmental behaviors. Though these factors would contribute to greater comprehensiveness, we have excluded them to lower the complexity of the estimation model in this study. Nonetheless, the extended TPB model provides an efficient workflow and valid estimates to answer our research question on the motivations versus hesitancies of Chinese students to visit UGSs after the easing of COVID-19 lockdown.

## Figures and Tables

**Table 1:** Socio-demographic profiles of respondents.

Variable	Category	Proportion
Gender	Male	55.92%
	Female	44.08%
Age	≤15	5.58%
	16–18	44.49%
	19–24	49.42%
	≥25	0.49%
Province of hometown	Jiangsu	67.92%
	Hunan	22.69%
	Beijing	4.93%
	Shanghai	3.61%
	Others	0.82%
Education level	Junior school and below	2.27%
	Senior high school	42.68%
	Undergraduate	48.23%
	Graduate	6.82%
Ethnic group	Han	95.58%
	Tujia	1.80%
	Hui	1.31%
	Zhuang	0.82%
	Others	0.49%
Frequency of visiting urban green park	Almost every day	6.44%
	Several times a week	12.12%
	Several times a month	24.75%
	Several times a year	27.02%
	Rarely	29.67%

**Table 2:** Indexes for model goodness to fit.

TLI	CFI	RMSEA
0.919 ***	0.931 ***	0.075 ***

***: *p* ≤ 0.01.

**Table 3:** Estimation results of the SEM.

Variables	Estimate	*p*-Value
Attitude toward the green park		
ATT1	0.872	0.000
ATT2	0.925	0.000
ATT3	0.817	0.000
Perceived behavior control		
PBC1	0.731	0.000
PBC2	0.752	0.000
PBC3	0.783	0.000
Subjective norms		
SN1	0.608	0.000
SN2	0.678	0.000
SN3	0.726	0.000
Perceived knowledge of COVID-19		
PK1	0.906	0.000
PK2	0.821	0.000
PK3	0.945	0.000
PK4	0.852	0.000
Risk perception		
RP1	0.728	0.000
RP2	0.834	0.000
RP3	0.788	0.000
RP4	0.788	0.000
Behavioral intention		
BI1	0.706	0.000
BI2	0.934	0.000
BI3	0.834	0.000

**Table 4:** The modeling results on seven hypothetical paths.

Hypothetical Paths			Coefficients	*p*-Value
H1: Behavioral intention	** ← **	Attitude	0.284	0.000
H2: Behavioral intention	** ← **	Perceived Behavior control	0.448	0.000
H3: Behavioral intention	** ← **	Subjective norms	0.239	0.000
H4: Perceived Behavior control	** ← **	Attitude	0.567	0.000
H5: Perceived Behavior control	** ← **	Subjective norms	0.379	0.000
H6: Subjective norms	** ← **	Risk perception	0.706	0.000
H7: Attitude	** ← **	Perceived knowledge	0.398	0.000

## Appendix A

**Table A1:** Measurement items for research constructs in questionnaire.

**Attitude toward the behavior**	
ATT1	I will often go to the surrounding greenspace to relax and relieve the pressure after the epidemic.
ATT2	I think that visiting urban green space can increase my contact with the outside world and have more social opportunities.
ATT3	I would like to recommend people around me visit urban green space after the epidemic.
**Perceived behavioral control**	
PBC1	Whether I visit urban green space is entirely up to me.
PBC2	Even if the number of tourists in the urban green parks is limited every day and need an appointment in advance, I would like to visit urban green space after the epidemic.
PBC3	I have sufficient resources, time, and opportunities to visit urban green space after the end of the epidemic.
**Subjective norms**	
SN1	My family and friends hope that I can visit the surrounding urban green parks for relaxation after the epidemic.
SN2	I will choose the urban green space with relatively few people to play after the epidemic.
SN3	I only want to go to the nearest urban green space, not to the suburbs, outside the city or other provinces after the epidemic.
**Perceived knowledge**	
PK1	I understand the source of COVID-19.
PK2	I know how to take measures to prevent COVID-19.
PK3	I often receive information about COVID-19 and health advice through the Internet and other media.
PK4	Compared with the average person, I know more facts about COVID-19.
**Psychological Risk**	
RP1	After the epidemic, I will worry about the risk of virus infection when I visit the surrounding urban green space.
RP2	After the epidemic, when I go to the urban green space, I will worry about safety because there are few people.
RP3	I am worried that the environmental quality and sanitation of green parks in surrounding cities are not ideal after the epidemic.
RP4	After the epidemic, I am worried that it will take a lot of time for the relevant health examination before entering the park.
**Behavioral intention for safer destinations**	
BI1	I plan to visit urban green space in the recent week.
BI2	I will exert effort to visit urban green space in the recent mouth.
BI3	I am willing to visit urban green space this year.

Note: All measurement items were evaluated with a seven-point scale, from “strongly disagree” (1) to “strongly agree” (7).
